# Treatment with d-penicillamine or zinc sulphate affects copper metabolism and improves but not normalizes antioxidant capacity parameters in Wilson disease

**DOI:** 10.1007/s10534-013-9694-3

**Published:** 2013-12-25

**Authors:** Gromadzka Grażyna, Karpińska Agata, Przybyłkowski Adam, Litwin Tomasz, Wierzchowska-Ciok Agata, Dzieżyc Karolina, Chabik Grzegorz, Członkowska Anna

**Affiliations:** 1 Second Department of Neurology, Institute of Psychiatry and Neurology, Sobieskiego 9, 02-957 Warsaw, Poland; 2 Department of Experimental and Clinical Pharmacology, Medical University of Warsaw, Warsaw, Poland

**Keywords:** Antioxidants, Copper, d-Penicillamine, Glutathione peroxidase, Oxidative stress, Treatment, Wilson disease, Zinc sulphate

## Abstract

Copper accumulation in tissues due to a biallelic pathogenic mutation of the gene: *ATP7B* results in a clinical phenotype known as Wilson disease (WD). Aberrations in copper homeostasis can create favourable conditions for superoxide-yielding redox cycling and oxidative tissue damage. Drugs used in WD treatment aim to remove accumulated copper and normalise the free copper concentration in the blood. In the current study the effect of decoppering treatment on copper metabolism and systemic antioxidant capacity parameters was analyzed. Treatment naïve WD patients (TNWD) (*n* = 33), those treated with anti-copper drugs (TWD) (*n* = 99), and healthy controls (*n* = 99) were studied. Both TNWD and TWD patients characterised with decreased copper metabolism parameters, as well as decreased total antioxidant potential (AOP), glutathione (GSH) level, activity of catalase, glutathione peroxidase (GPx), and S-transferase glutathione, compared to controls. TWD patients had significantly lower copper metabolism parameters, higher total AOP and higher levels of GSH than TWD individuals; however, no difference was observed between these two patient groups with respect to the rest of the antioxidant capacity parameters. Patients who had undergone treatment with d-penicillamine or zinc sulphate did not differ with respect to copper metabolism or antioxidant capacity parameters, with the exception of GPx that was lower in d-penicillamine treated individuals. These data suggest that anti-copper treatment affects copper metabolism as well as improves, but does not normalize, natural antioxidant capacity in patients with WD. We propose to undertake studies aimed to evaluate the usefulness of antioxidants as well as selenium as a supplemental therapy in WD.

## Introduction

Wilson disease (WD; Online Mendelian Inheritance in Man [OMIM] accession number #277900) is an autosomal recessive copper storage disease resulting from biallelic pathogenic mutation in the *ATP7B* gene, which encodes the P-type adenosine triphosphatase (ATPase), ATPase-7B (OMIM number *606882) (Ala et al. 2007). The physiological functions of ATPase-7B include the intracellular translocation of copper to the Golgi apparatus where it is incorporated into apoceruloplasmin, and the transport of excess copper to secretory vesicles for its excretion into the biliary canaliculi (Terada et al. 1998; Fatemi and Sarkar 2002). In people with pathogenic mutations in both alleles of *ATP7B* severe dysfunction of *ATP7B* results in excessive copper accumulation primarily in the hepatocytes and in the brain (Mercer 2001; Petrukhin et al. 1994; Brewer 2005), thus affected individuals primarily manifest with hepatic and/or neurological symptoms that may appear in childhood, adulthood, or more rarely in the elderly (Brewer 2000; Riordan and Williams 2001; Ferenci et al. 2007).

Several mechanisms have been proposed to explain copper-induced cellular toxicity. The basis for most theories is the ability of copper to participate in the formation of reactive oxygen species (Lynch and Frei 1993). In the presence of the superoxide anion radical or other reducing agents, copper^2+^ can be reduced to copper^+^, which is capable of catalysing the formation of hydroxyl radicals from hydrogen peroxide via the Fenton reaction (Ercal et al. 2001; Gaetke and Chow 2003). Thus, the oxidative stress associated with copper accumulation may be a central mechanism underlying the deleterious effects of WD. Some previous reports have confirmed such hypothesis, including recent in vitro study on hepatic stellate cells (Xu et al. 2013) and experimental study on Long-Evans Cinnamon rats (Yamamoto et al. 2001), as well as clinical studies: one that enrolled 13 WD children, demonstrating that more severe liver damage is associated with decreased antioxidant enzymes in the liver of untreated WD children (Nagasaka et al. 2006), and another one that reported a decreased total serum antioxidant capacity in WD patients (Bruha et al. 2012).

There are few drugs available for the treatment of WD, including d-penicillamine, trientine, and zinc salts. d-penicillamine has a free sulfhydryl group, which functions as a copper chelating moiety. The major effect of d-penicillamine in WD is to promote the urinary excretion of copper. Trientine (triethylene tetramine dihydrochloride) has a polyamine-like structure that allows copper binding by forming a stable complex with the four constituent nitrogens in a planar ring. Like penicillamine, trientine promotes copper excretion in the urine. The mechanism of action of zinc salts depends on interfering with the uptake of copper from the gastrointestinal tract (Ala et al. 2007; brewer 2005; Roberts and Schilsky 2008).

As anti-copper drugs aim to remove accumulated copper and normalize the free copper concentration in the blood to reverse copper poisoning, it may be hypothesized that such treatment may diminish oxidative stress induced by copper overload among WD patients. It has been suggested that the antioxidant profile in tissues or in biological fluids may be helpful for assessing oxidative stress in humans (Polidori et al. 2001; Ghiselli et al. 2000). Thus, in the current study, we aimed to evaluate the effect of anti-copper treatment on systemic antioxidant capacity of WD patients by comparing the systemic antioxidants, as well as total antioxidant potential (AOP) between treatment naïve WD patients (TNWD), patients on anti-copper treatment with d-penicillamine or zinc sulphate (TWD), and healthy controls.

## Patients and methods

### Patients

The clinical and laboratory data for 33 consecutive patients newly diagnosed with WD who had never been treated with decoppering drugs (TNWD), as well as 99 patients who had undergone treatment with anti-copper drugs (50 with D-penicillamine, 49 with zinc sulphate; TWD), were evaluated and analysed at the Second Department of Neurology, Institute of Psychiatry and Neurology in Warsaw, Poland. The median time of treatment was 48.5 months (IQR 47.0; range: 6–168). All patients met the WD diagnostic criteria of the 8th International Conference of Wilson Disease and Menkes Disease (Ferenci et al. 2003). The baseline characteristics of the evaluated groups of patients are given in Table 1. The control group included 99 age- and sex-matched healthy volunteers with a mean age of 35.6 ± 10.5 years, who did not have a family history of WD, diagnosed liver disease, neurological or psychiatric disease, chronic inflammatory disease, or infectious disease. The control subjects were recruited from hospital staff and their family. The three most frequent *ATP7B* mutations in the Polish population (Gromadzka et al. 2005) were absent in these individuals as documented by DNA sequence analysis using previously described methods (Gromadzka et al. 2010).

**Table 1:** Baseline characteristics of WD patients

Characteristics	Treatment naïve patients (*n* = 33)	Patients on anti-copper treatment (*n* = 99)	Statistics
Male/female			
No. of patients	20/13	40/59	*χ* ^2^ 4.07; df1; *P* < 0.05
Age			
mean ± SD	36.7 ± 11.3	34.9 ± 10.3	*P* < 0.40
The clinical form of the WD			
No. of patients (%)			
Hepatic	15 (45.4)	31 (32.6)	*χ* ^2^ 0.22; df 1; *P* < 0.65
Neurological	18 (54.5)	64 (67.4)	
No. of presymptomatic	–	4	
Decoppering treatment			
No. of patients			
d-penicillamine (Cuprenil)	**–**	50	**–**
Zinc sulphate (Zincteral)	**–**	49	**–**
Duration of treatment (months)			
median (IQR)	**–**	48.5 (47.0)	**–**
d-penicillamine (Cuprenil)		42.0 (38.0)	*P* < 0.08
Zinc sulphate (Zincteral)		58.0 (54.0)	

*IQR* interquartile range; *SD* standard deviation

Blood samples were collected prospectively from all study subjects for laboratory analysis of oxidative stress parameters between 2010 and 2012. All groups of individuals were examined in the same time schedule. The biological material was collected as separated aliquots to be used in individual measurements to avoid repeated freeze–thaw cycles. The samples were stored at −70 °C. The samples were applied to the assays immediately after thawing to avoid decrease of enzyme activity. All study participants provided written informed consent to participate in this study. The study protocol was approved by the local Ethics Committee and conforms to the ethical guidelines of the 1975 declaration of Helsinki.

### Laboratory analyses

#### Copper metabolism parameters

Serum ceruloplasmin was measured by a colorimetric enzymatic assay as previously described (Ravin 1961). Serum copper was determined by atomic adsorption spectroscopy.

#### Systemic antioxidant capacity and selected small and large molecule antioxidants

Serum total AOP was evaluated using OxisResearch kit AOP-450 (cat. no. 21053; Biokom Company, Warsaw, Poland). The principle of this test depends on the measuring the reduction potential of the sample that converts copper^2+^ to copper^+^, thus changing the ion’s absorption characteristics. The reduced form of copper selectively forms a stable 2:1 complex with the chromogenic reagent with an absorption maximum at ca. 450 nm. A known concentration of Trolox is used to create a calibration curve, with the data being expressed as μM copper reducing equivalents. Serum manganese superoxide dismutase (MnSOD) activity was investigated using the Superoxide Dismutase Assay Kit (cat. no. 706002; Cayman Chemical Company; Ann Arbor, MI, USA) with an assay range of 0.025–0.25 U/mL. Serum glutathione peroxidase (GPx) activity was investigated using theGPx Assay Kit (cat. no. 703102; Cayman Chemical Company) with an assay range of 50.0–344.0 nmol/min/mL. Plasma glutathione *S*-transferase (STG) activity was investigated using the glutathione *S*-transferase Assay Kit (cat. no. 703302; Cayman Chemical Company). Serum catalase (Cat) activity was investigated using the Catalase Assay Kit (cat. no. 707002; Cayman Chemical Company) with an assay range of 2.0–35.0 nmol/min/mL. Plasma glutathione (GSH) concentration was investigated using the Glutathione Assay Kit (cat. no. 703002; Cayman Chemical Company) with an assay range of 0.0–16.0 μM.

### Statistical analysis

The normality of the analysed continuous variables was determined using Kolmogorov–Smirnov and Lilliefors tests. Data were presented as mean and standard deviation if normally distributed, or median and interquartile range if the variables were not normally distributed.

A Student’s *t* test was used to compare two groups of normally distributed data.

The Mann–Whitney U-test was used for comparisons of variables that were not normally distributed. Qualitative data are described as ratios with percentages. Comparisons between groups were performed using the *χ* ^2^ with Yates correction, if appropriate, or the Fisher exact test when the hypothesis for the *χ* ^2^ was not fulfilled. Significance was set at *P* < 0.05. All statistical analyses were performed using STATISTICA 10.0 software (StatSoft PL, Cracow, Poland).

## Results

### Patient characteristics

The mean age of TNWD patients was 36.7 ± 11.3 years, which was not significantly different from the age of control subjects (33.5 ± 5.9 years, *P* = 0.09). The two groups did not significantly differ in regards to gender (*χ* ^2^ 0.003, df 1, *P* < 0.95). The mean age of TWD patients (34.8 ± 10.3 years) and their gender was not different from the age and gender of controls (*P* < 0.07; *χ* ^2^ 2.26, df 1, *P* < 0.07, respectively). TNWD and TWD patients did not differ with respect to age, but the TWD was group characterized by a higher prevalence of females (Table 1).

### Copper metabolism parameters

TWD and TNWD patients had significantly lower serum copper and ceruloplasmin levels than controls. TWD individuals had significantly lower values of copper metabolism parameters than those from the TNWD group (Table 2).

**Table 2:** Copper metabolism and antioxidant capacity parameters among WD patients and healthy controls

Parameter	Controls (*n* = 99)	WD patients treatment naive (*n* = 33)	WD patients on anti-copper treatment (*n* = 99)	*P* value*
Copper metabolism				
Serum copper, μg/dL, median (IQR)	91.0 (24.0)	63.0 (17.0)	23.0 (35.0)	a < 0.001 b < 0.001 c < 0.001
Serum ceruloplasmin, mg/dL, median (IQR)	31.8 (10.7)	15.3 (8.0)	5.4 (7.3)	a < 0.001 b < 0.001 c < 0.001
Antioxidant capacity				
Total antioxidant capacity, CREs, mean ± SD	848.1 ± 130.0	652.5 ± 151.7	772.4 ± 132.9	a < 0.001 b < 0.001 c < 0.001
Glutathione, μmol/L, median (IQR)	2.2 (2.1)	0.0 (0.9)	1.0 (2.1)	a < 0.001 b < 0.001 c < 0.007
S-transferase glutathione, nmol/min/mL, median (IQR)	17.6 (7.8)	9.7 (6.9)	13.5 (14.2)	a < 0.001 b < 0.001 c < 0.141
Glutathione peroxidase, nmol/min/mL, mean ± SD	96.1 ± 16.5	81.9 ± 26.6	72.2 ± 28.3	a < 0.010 b < 0.001 c < 0.108
Catalase, nmol/min/mL, mean ± SD	178.4 ± 78.1	128.6 ± 78.7	142.2 ± 69.5	a < 0.001 b < 0.001 c < 0.393
Manganese superoxide dismutase, U/mL, median (IQR)	14.4 (5.7)	13.5 (3.6)	12.8 (5.0)	a < 0.270 b < 0.016 c < 0.496

Normal ranges: 25.0–45.0 mg/dL for serum ceruloplasmin; 70.0–140.0 μg/dL for serum Cu CREs, μM Cu reducing equivalents

*IQR* interquartile range; *SD* standard deviation

*a, *P* for the comparison between WD patients treatment naive and controls; b, *P* for the comparison between WD patients on anti-Cu treatment and controls; c, *P* for the comparison between WD patients treatment naive and on anti-Cu treatment

### Antioxidant capacity parameters

Both TWD and TNWD patients were characterised by significantly decreased blood levels of total AOP, GSH, and activity of Cat, GPx, compared to controls. TNWD patients also had decreased activity of MnSOD (Table 2). TWD patients had significantly higher total blood AOP and higher levels of GSH than TWD individuals; however, no difference was observed between these two patient groups with respect to the rest of the antioxidant capacity parameters (Table 2).

### Copper metabolism, antioxidant capacity parameters and the type of anti-copper treatment

TWD patients who had undergone treatment with d-penicillamine or zinc sulphate did not differ with respect to all antioxidant capacity parameters, with the exception of GPx that was significantly lower in d-penicillamine treated individuals (Table 3).

**Table 3:** Copper metabolism and antioxidant capacity parameters among WD patients treated with zinc sulphate or d-penicillamine

Parameter	Zinc sulphate penicyl (*n* = 49)	d-penicillamine (*n* = 50)	*P*
Copper metabolism			
Serum copper, μg/dL median (IQR)	23.0 (35.0)	21.0 (32.0)	<0.787
Serum ceruloplasmin, mg/dL median (IQR)	5.6 (5.5)	5.3 (11.7)	<0.649
Antioxidant capacity			
Total AOP, CREs mean ± SD	749.7 ± 128.9	795.1 ± 134.2	<0.091
Serum glutathione, μmol/L, median (IQR)	1.2 (1.6)	0.7 (2.4)	<0.738
S-transferase glutathione, nmol/min/mL, median (IQR)	15.0 (14.2)	12.3 (13.4)	<0.260
Glutathione peroxidase, nmol/min/ml, mean ± SD	87.6 ± 29.6	57.3 ± 17.0	<0.001
Catalase, nmol/min/ml, mean ± SD	142.1 ± 63.2	143.3 ± 76.2	<0.930
Manganese superoxide dismutase, U/ml, median (IQR)	12.3 (5.8)	12.9 (3.2)	<0.238

Normal ranges: 25.0–45.0 mg/dL for serum ceruloplasmin; 70.0–140.0 μg/dL for serum Cu

*CREs* μM Cu reducing equivalents; *IQR* interquartile range; *SD* standard deviation

### Antioxidant capacity parameters: correlation to copper metabolism, age at testing and duration of anti-copper treatment

In TNWD and TWD patients, most antioxidant capacity parameters did not correlate with copper metabolism parameters (Table 4). Activity of GPx negatively or positively correlated with serum copper and ceruloplasmin in patients receiving zinc sulphate or d-penicillamine, respectively. MnSOD negatively correlated with serum copper in the d-penicillamine treated group. The duration of treatment with zinc sulphate did not correlate with copper metabolism or antioxidant capacity parameters (Table 5). A longer duration of treatment with d-penicillamine correlated with higher serum ceruloplasmin and higher blood activity of STG (Table 4).

**Table 4:** Correlation between serum copper and ceruloplasmin and antioxidant capacity in WD patients

Parameter	Serum copper			Serum ceruloplasmin		
	Patients treatment naive	Patients on treatment		Patients treatment naive	Patients on treatment	
		Zinc sulphate	d-penicillamine		Zinc sulphate	d-penicillamine
Total AOP	−0.29	0.03	0.07	0.04	−0.10	−0.02
Glutathione	−0.03	0.17	0.05	0.25	0.20	0.17
S-transferase glutathione	−0.14	−0.02	0.05	0.10	0.01	0.24
Glutathione peroxidase	−0.14	−0.43*	0.34*	0.04	−0.41*	0.36*
Catalase	−0.14	−0.08	−0.06	0.14	−0.13	0.08
Manganese superoxide dismutase	0.04	−0.20	−0.32*	0.32	−0.03	−0.22

Data are shown as correlation coefficients

*AOP* antioxidant potential

*P < 0.05

**Table 5:** Correlation between duration of treatment and copper metabolism and antioxidant capacity parameters

Parameter	WD patients on treatment	
	Zinc sulphate	d-penicillamine
Copper metabolism		
Serum copper	0.18	0.17
Serum ceruloplasmin	0.13	0.32*
Antioxidant capacity		
Total AOP	−0.04	0.18
Glutathione	0.09	0.05
S-transferase glutathione	0.11	0.29*
Glutathione peroxidase	−0.29	0.04
Catalase	0.21	0.24
Manganese superoxide dismutase	−0.08	−0.04

Data are shown as correlation coefficients

*AOP* antioxidant potential

* *P* < 0.05

## Discussion

Discoveries over the last few years suggest that oxidative damage may play an important role in the pathogenesis of tissue damage in many pathological conditions, including liver and brain diseases. WD is a special case characterised by copper storage-induced progressive liver impairment that in about 40 % of patients is associated with brain disease. As copper is a redox active metal, aberrations in its’ homeostasis may create favourable conditions for superoxide-yielding redox cycling and oxidative damage to susceptible regions within the liver and brain, where easily oxidised substrates abound (e.g. membrane polyunsaturated lipids), and redox-based neurochemical reactions occur. Some previous reports have suggested that oxidative stress may play an important role in the pathogenesis of tissue damage in WD (Yamamoto et al. 2001; Nagasaka et al. 2006; Bruha et al. 2012; Xu et al. 2013).

Our study evaluated the effect of anti-copper treatment with d-penicillamine or zinc sulphate on copper metabolism and on systemic antioxidant capacity parameters in WD patients. Our observations suggest that copper metabolism disturbance in WD is associated with significant changes in systemic antioxidant capacity parameters in a direction favouring enhanced oxidative stress. Moreover, our data suggest that while anti-copper treatment affects copper metabolism and improves systemic antioxidant capacity, it does not normalize it. In the current study TWD patients had significantly lower copper metabolism parameters, blood GSH concentrations, activity of the antioxidant enzymes: Cat, MnSOD, GPx and STG, and total AOP compared with healthy controls (Table 2). The duration of treatment and copper metabolism parameters did not, or was only weakly correlated with antioxidant capacity parameters. TWD individuals had significantly lower copper metabolism parameters, higher total AOP, and higher blood GSH, compared with patients who had never been treated with anti-copper drugs, but no difference was observed for the remaining antioxidant capacity parameters (Tables 4, 5).

Our results suggest that patients treated with d-penicillamine or zinc sulphate do not differ for copper metabolism parameters nor for most antioxidant capacity parameters studied. However, patients treated with d-penicillamine had a significantly lower GPx activity (57.3 ± 17.0 nmol/min/mL) than those treated with zinc sulphate (87.6 ± 29.6 nmol/min/mL), patients treatment naïve (81.9 ± 26.6 nmol/min/mL), as well as healthy controls (96.1 ± 16.5 nmol/min/mL) (*P* < 0.001 for all the comparisons).

Glutathione peroxidase (GPx is one of the primary antioxidant enzymes protecting cells against oxidative stress (Arthur 2000; Brigelius-Flohé and Maiorino 2013). It is regarded as a crucial enzyme involved in reducing damaging hydrogen peroxide and lipid/phospholipids peroxides (Spallholz 1990; Bor et al. 1999). Impaired activity of GPx may thus result in membrane lipid peroxidation that is an important factor contributing to cellular damage (Kühn and Borchert 1987; Ursini and Bindoli 1987). Our observation of decreased GPx activity in patients treated with d-penicillamine is in agreement with reports by (Chaudiere et al. 1984; Chaudiere and Tappel 1984), who documented that d-penicillamine is a potent in vitro inhibitor of GPx. It has also been shown that GPx activity is decreased in vitamin E-deficient chickens treated with high doses of d-penicillamine (Mercurio and Combs 1985). Given that in the presence of copper ions d-penicillamine may undergo auto-oxidation and produce toxic hydrogen peroxide (Joyce 1991; Starkebaum and Root 1993), it seems possible that in the settings of decreased GPx activity, high amounts of free radicals may be produced via the Haber–Weiss and Fenton reactions, leading to oxidative cellular damage. In this context, our observation may supply an explanation for a wide variety of d-penicillamine toxicities that had been widely described by Roberts and Schilsky in (2008).

An important point to address here are possible therapies that could be used to reverse the effect of d-penicillamine on GPx activity. In this regard, dietary supplementation with selenium may protect patients from this particular side effect of d-penicillamine treatment. Selenium is contained within the active center of GPx, and is necessary for the expression and activity of this enzyme (Flohe et al. 1973; Roy et al. 2005). Selenium supplementation has been shown to increase GPx activity in various experimental models (Sappey et al. 1994; Makropoulos et al. 1996), as well as in human diseases (Schnabel et al. 2008; Al-Taie et al. 2003; Boškovic et al. 2011).

Our study has some limitations. Because it was a clinical study, and not an experimental study, we were unable to evaluate the effect of different doses of zinc sulphate or d-penicillamine on antioxidant capacity parameters. We also did not correlate antioxidant capacity parameters with changes in the clinical status of patients—this is something that should be evaluated prospectively including both short-term as well as long-term evaluation of antioxidant capacity.

In conclusion, our study suggests that despite anti-copper treatment, WD patients have abnormalities in copper metabolism as well as decreased antioxidant capacity and thus are at increased risk of developing various pathological conditions related to oxidative stress. In this regard, molecules with antioxidant activity (like melatonin, lipoic acid, coenzyme Q10, vitamin E, vitamin C, *N*-acetyl cysteine, and others) may be beneficial as a supplementary treatment in WD (Boškovic et al. 2011). Future studies aimed to evaluate the possible usefulness of antioxidants as a supplemental therapy in this disease are warranted. In regards to our observation of impaired activity of GPx in patients treated with d-penicillamine, studies aimed to assess the possible benefit of dietary supplementation with selenium should also be performed.
